# EHMTI-0303. Treatment of medication overuse headache

**DOI:** 10.1186/1129-2377-15-S1-C41

**Published:** 2014-09-18

**Authors:** SK Madsen, SB Munksgaard, T Wienecke

**Affiliations:** 1 Department of Neurology Glostrup Hospital, Danish Headache Center, Copenhagen, Denmark

## Background

Medication overuse headache (MOH) has an estimated prevalence of 0.7-1.7 % on a global scale. It is highly invalidating compared with other chronic headaches and has great socioeconomic costs. Many different treatment initiatives are in use today, but few are based on scientific evidence.

## Aim

To review and evaluate currently used treatment options of MOH.

## Method

A simple search using MeSH term "medication overuse headache", previously used terms "Rebound headache" and "drug-induced headache" and "headache" and drug misuse" was made. Only clinical trials (n=41) were included. Predictors of outcome were collected from different studies and compared.

## Results

Treatment outcome was not different between patients treated with advice alone, outpatient, or inpatient treatment. Stratifying patients in groups with or without comorbidities revealed a difference in treatment outcome for patients with comorbidities in favor of inpatient treatment. Different prophylactic medication given during medication overuse showed a significant reduction in headache frequency for topiramate, gabapentin and pregabalin, but not for prednisolon compared with placebo. Delaying prophylactic medication to after detoxification reduced the number of patients who needed prophylactic medication. Predictors of treatment outcome were ambigous.

## Conclusion

The type of treatment (inpatient, outpatient or advice alone) should be selected according to comorbidities of the patient. Delayed initiation of prophylactic medication probably causes less need of prophylactic medication and betters the effect of subsequent prophylactic medication. Topiramate, gabapentin and pregabalin are useful as prophylactics for MOH patients who are unable to undergo detoxification.

No conflict of interest.

